# Fast GPU-Based Generation of Large Graph Networks From Degree Distributions

**DOI:** 10.3389/fdata.2021.737963

**Published:** 2021-11-26

**Authors:** Maksudul Alam, Kalyan Perumalla

**Affiliations:** Computer Science and Mathematics Division, Oak Ridge National Laboratory, Oak Ridge, TN, United States

**Keywords:** SIMT architectures, graph generation, GPU (graphic processing unit), random network, large graph

## Abstract

Synthetically generated, large graph networks serve as useful proxies to real-world networks for many graph-based applications. The ability to generate such networks helps overcome several limitations of real-world networks regarding their number, availability, and access. Here, we present the design, implementation, and performance study of a novel network generator that can produce very large graph networks conforming to any desired degree distribution. The generator is designed and implemented for efficient execution on modern graphics processing units (GPUs). Given an array of desired vertex degrees and number of vertices for each desired degree, our algorithm generates the edges of a random graph that satisfies the input degree distribution. Multiple runtime variants are implemented and tested: 1) a uniform static work assignment using a fixed thread launch scheme, 2) a load-balanced static work assignment also with fixed thread launch but with cost-aware task-to-thread mapping, and 3) a dynamic scheme with multiple GPU kernels asynchronously launched from the CPU. The generation is tested on a range of popular networks such as Twitter and Facebook, representing different scales and skews in degree distributions. Results show that, using our algorithm on a single modern GPU (NVIDIA Volta V100), it is possible to generate large-scale graph networks at rates exceeding 50 billion edges per second for a 69 billion-edge network. GPU profiling confirms high utilization and low branching divergence of our implementation from small to large network sizes. For networks with scattered distributions, we provide a coarsening method that further increases the GPU-based generation speed by up to a factor of 4 on tested input networks with over 45 billion edges.

## 1 Introduction

### 1.1 Motivation

Random graph networks sometimes serve as useful proxies in modeling complex systems. To aid in such use, network generation algorithms are employed to create random network instances on demand (Penschuck et al., 2020). When the scale of the studied system is large (such as the Internet (Faloutsos et al., 1999; Siganos et al., 2003), biological networks (Girvan and Newman, 2002), and social networks (Leskovec, 2008; Kwak et al., 2010; Yang and Leskovec, 2015)), the generation algorithms need to be carefully designed and implemented to increase the speed of generation. Also, to accurately mimic the desired properties of a targeted network of interest, the generated proxies need to preserve those properties. Degree distribution is one of the prominent properties by which different network types are characterized. Therefore, generation of random networks conforming to desired degree distributions is important in network applications.

Degree distributions may be specialized or general in nature, and the network generators vary depending on the type of degree distribution of interest. In the past, a few well-understood graph models have been developed to capture the diversity of the degree distributions in the generated network. These include Erdős–Rényi (Erdős and Rényi, 1960), stochastic block models (Holland et al., 1983), small-world (Watts and Strogatz, 1998), Barabási–Albert (Barabási and Albert, 1999; Albert et al., 2000), exponential random graph (Frank and Strauss, 1986; Robins et al., 2007), recursive matrix (Chakrabarti et al., 2004), stochastic Kronecker graph (Leskovec and Faloutsos, 2007; Leskovec, 2010), and HOT (Carlson and Doyle, 1999) models. Each of these models have been developed considering some specific aspects of the networks. Many of these models generate graphs with a pre-defined class of degree distributions. Here, we focus on general degree distributions in which any desired set of degrees can be specified as input.

### 1.2 Efficient Algorithms for Graph Generation

As the scale of the network increases in terms of the number of vertices and edges, the time taken to generate the network also increases. Therefore, generation of large random graphs necessitates efficient algorithms, in terms of both time and space requirements. However, even efficient sequential algorithms for generating such graphs were not prevalent until recently. While some efficient sequential algorithms have emerged (Chakrabarti et al., 2004; Batagelj and Brandes, 2005; Leskovec and Faloutsos, 2007; Miller and Hagberg, 2011), these algorithms can generate graphs with only millions of vertices in a reasonable amount of time. Without efficient realization of the generator on the computational platform, the generation of graphs with billions of vertices can take a long amount of computational time.

Advancements in computing hardware, software, and algorithms have enabled increasing levels of variety, sophistication and scale of generated graph networks. On conventional processors, some of the early algorithms included efficient sequential generators of Erdős–Rényi and Barabási–Albert networks (Batagelj and Brandes, 2005), and a distributed memory–based parallel algorithm to generate networks with approximate power–law degree distribution (Yoo and Henderson, 2010). Recent work has also developed distributed memory–based parallel algorithms to generate exact power–law degree distributions (Alam et al., 2013; Meyer and Penschuck, 2016; Sanders and Schulz, 2016). A shared–memory-based parallel algorithm has been designed for generating networks with power–law degree distribution (Azadbakht et al., 2016), and another massively parallel network generator based on the Kronecker model is available (Kepner et al., 2018). Highly scalable generators for Erdős-Rényi, 2D/3D random geometric graphs, 2D/3D Delaunay graphs, and hyperbolic random graphs are now known (Funke et al., 2019). R-MAT (Chakrabarti et al., 2004) and stochastic Kronecker graph (SKG) (Leskovec, 2010) are some popular models to generate networks with power–law degree distribution using matrix multiplication. The SKG model is notable in that the Graph500 group chose the SKG model in their supercomputer benchmark due to its simplicity of implementation.

In our previous work (Alam et al., 2016), we have shown that a generalized and efficient generation of degree distribution-conforming networks is possible using an approach based on the Chung-Lu (CL) model (Chung and Lu, 2002a; Chung and Lu, 2002b). The model is suitable for generating proxy networks from the degree distribution of nearly any real-world network. The CL model is remarkable due to its similarity to the SKG model (Pinar et al., 2011). In fact, the CL model can be used to not only replace the SKG model, but also expand the generation to an even wider range of degree distributions. An efficient sequential algorithms for the CL model is available (Miller and Hagberg, 2011), as also a distributed–memory parallel algorithm (Alam and Khan, 2015). An efficient and scalable algorithmic method to generate Chung–Lu, block two–level Erdős–Rényi (BTER), and stochastic blockmodels have been previously presented by us (Alam et al., 2016).

Although there has been progress in scalable generation on conventional processor (CPU) systems, no algorithms have so far been presented in the literature to exploit specialized accelerated hardware that offers significantly faster computational possibilities.

### 1.3 Graphics Processing Units-Based Network Generation

In accelerated computing, graphics processing units (GPUs) represent a cost-effective, energy-efficient, and widely available parallel processing platform. GPUs are highly parallel, multi-threaded, many-core processors that have greatly expanded beyond graphics operations and are now widely used for general purpose computing. Most desktops, laptops and workstations contain this next generation computing based on GPUs. They are now so prevalent that many high performance computing and supercomputing systems are also built using GPU hardware as the major computational workhorse. However, conventional CPU-oriented algorithms are not ported easily to GPU platforms. The unique execution mode of GPUs needs to be carefully exploited to realize their promise of computational speed.

The use of GPUs is prevalent in many areas such as scientific computation, complex simulations, big data analytics, machine learning, and data mining. Although GPUs are now being applied to graph problems, there is a general lack of GPU-based network generators. Some of the known works include a GPU–based algorithm for generating Erdős–Rényi networks (Nobari et al., 2011) and a GPU–based algorithm for generating random networks (Leis et al., 2013) using the small–world model (Watts and Strogatz, 1998). However, until recently no GPU–based algorithm existed for other important degree distributions such as power–law. In our previous research, we presented the first GPU-based algorithms to generate networks with power–law degree distributions (Alam, 2016; Alam et al., 2016; Alam and Perumalla, 2017a; Alam and Perumalla, 2017b), as well as a multi-GPU implementation for the same problem (Alam et al., 2019). So far, to the best of our knowledge, there is no GPU-based algorithm to generate networks conforming to arbitrary degree distributions.

### 1.4 Contributions and Organization

In this paper, we focus on achieving a GPU-based capability for fast generation of random networks conforming to any specified degree distribution. Our aim is achieve a high speed of graph generation by designing and implementing new algorithms specifically suited to the SIMT execution style required on GPUs. Towards this end, we present a novel GPU-based method, based on grouping the vertices by their degrees, that leads to space and time efficient algorithms.

### Our Main Contributions Are Summarized Below

1. The algorithm presented here is the first GPU-based algorithm published in the literature for degree distribution-based network graph generation.
2. To improve the performance of network generation on GPUs, we present a new distribution coarsening approach that provides gains in run time without affecting the degree distribution of the generated output graph.
3. The rate of network generation (measured in terms of millions of edges generated per second) achieved by our algorithm design and implementation (on a single CPU or GPU device) is among the highest reported so far in the literature, exceeding 50 billion edges per second for some test networks.

In Section 2, we recapitulate the basic concepts and the algorithmic building blocks for degree distribution-conforming graph generation, borrowing the terminology from our previous work that was based on CPU platforms (Alam et al., 2016). In Section 3, we build on this basic, generic framework and re-target it specifically to suit the SIMT (single instruction multiple thread) architecture of GPU accelerators. The GPU algorithms and task scheduling approaches are described in the same section. A detailed study of the runtime performance is presented in Section 4 using multiple test networks. Performance improvements are also reported using a degree distribution coarsening scheme designed to improve the task-to-thread mapping on the GPU architecture. The research is summarized and future work is identified in Section 5.

## 2 Theory and Algorithmic Approach

### 2.1 Problem and Solution Approach

The problem of generating a random network that conforms to a given degree distribution is defined as follows. The desired output is a graph *G*(*n*, *m*) of *n* vertices and *m* edges such that the degree of connectivity of each vertex conforms to a user-specified distribution. That is, given an input degree distribution, a random network is to be generated such that the edge connectivity of vertices of the generated network obeys the input degree distribution. The input could be specified either as a desired histogram of the vertex degrees in the graph, or it could be specified as the exact count of the connectivity degree for each vertex in the graph.

**Input**: The input in general is an array in which element *i* the number of neighbors *b*_*i*_ of the *i* ^th^ vertex. In other words, it is an array of *n* expected vertex degrees, denoting one degree count per vertex: $B=\left\{b_{1},b_{2},\ldots b_{n}\right\}$ , 0 ≤ *b*_*i*_ < *n*. Let $D=\left\{d_{1},d_{2},\ldots ,d_{\Lambda }\right\}$ be the set of all Λ distinct, non-zero degrees in B . Let *n*_*i*_ be the number of vertices each of which has an expected degree *d*_*i*_. Note that not all degrees need be present in the input distribution. In other words, *d*_*i*_ for which *n*_*i*_ = 0 are not included in the degree distribution. Thus,

$$\mathrm{DD}=\left\{\left(d_{i},n_{i}\right)|1\leq i\leq \Lambda \text{ and }0<d_{i}<n\text{ and }0<n_{i}\leq n\right\}$$ (1)

represents the input degree distribution, where $n=\sum_{i=1}^{\Lambda }n_{i}$ . Also, we denote by *S* the sum of the degrees of all vertices, that is, $S=\sum_{i=1}^{\Lambda }\left(d_{i}n_{i}\right)$ .

**Preprocessing**: Our algorithm accepts either the sequence of degrees B or the degree distribution DD as input. If B is specified as input, it is converted into its equivalent DD degree distribution. The vertices are grouped by their expected degrees: if $V_{i}=\left\{u|b_{u}=d_{i}\right\}$ is the group of vertices with expected degree *d*_*i*_, then *n*_*i*_ = |*V*_*i*_| is the number of vertices in *V*_*i*_ for 1 ≤ *i* ≤ Λ. Therefore, in the rest of this paper, without loss of generality, we assume the input is specified as DD .

**FIGURE 1:**
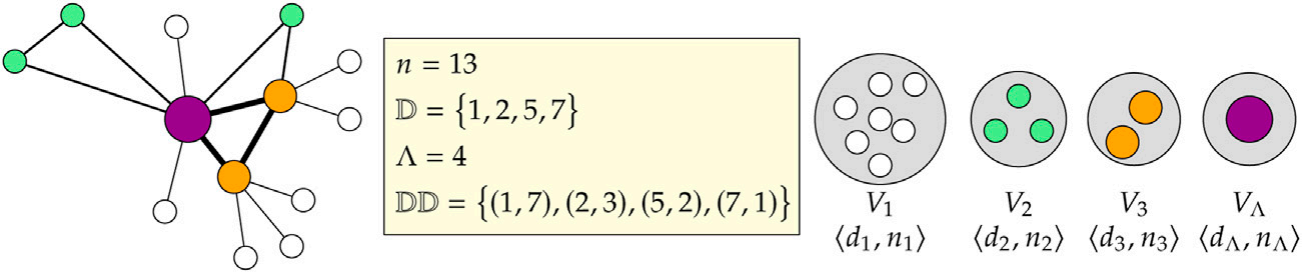
Illustrative example of the input and preprocessing for a desired graph with *n* = 13 vertices.

The terms are illustrated in Figure 1 with a small example graph comprising *n* = 13 vertices such that there are Λ = 4 unique degrees given as $D=\left\{1,2,5,7\right\}$ . The input degree distribution, therefore, is $\mathrm{DD}=\left\{\left(1,7\right),\left(2,3\right),\left(5,2\right),\left(7,1\right)\right\}$ . Therefore, there are four groups, *V*_1_…*V*_4_, containing 7, 3, 2, and 1 vertices respectively.

**Output**: With the preceding background, every edge *e* = (*u*, *v*) in the output graph will correspond to exactly one of the following two types:

1. **Intra-group edge**, or **intra edge** for short, is an edge between *u* and *v* if both *u* and *v* belong to the same group, that is, *u*, *v* ∈ *V*_*i*_ for some *i*, and

2. **Inter-group edge**, or **inter edge** for short, is an edge between *u* and *v* if *u* and *v* belong to two different groups, that is, *u* ∈ *V*_*i*_ and *v* ∈ *V*_*j*_ for some *i* ≠ *j*.

Problem We now redefine the graph generation problem to that of correctly and efficiently generating all the intra edges and inter edges. The union of the two sets of edges will directly constitute a graph network whose combined vertex-connectivity conforms to the desired degree distribution specified as input. In generating both types of edges, we exploit the Chung–Lu (CL) model in which any pair of vertices *u* and *v* are connected by an edge with the probability $p_{u,v}=\frac{b_{u}b_{v}}{S}$ , where *S* = *∑*_*u*_ *b*_*u*_ (assuming $\max_{u}b_{u}^{2}\leq S$ , we have 0 ≤ *p*_*u*,*v*_ ≤ 1 for all *u* and *v*) (Chung and Lu, 2002a; Chung and LU, 2002b). For simple graphs without self-loops (*u* ≠ *v*), the expected degree of a vertex *u* is $\sum_{v}\frac{b_{u}b_{v}}{S}=b_{u}-\frac{b_{u}^{2}}{S}$ , which converges to *b*_*u*_ for large graphs.

### 2.2 Vertex Labels

Each vertex is identified by a unique integer label from 1 to *n* as follows. Let *λ*_*i*_ be the label of the first vertex of a group *V*_*i*_, where *λ*_1_ = 1 and $\lambda_{i}=1+\sum_{j=1}^{i-1}n_{j}$ for *i* > 1. Then, the vertices in *V*_*i*_ are labeled by the integers from *λ*_*i*_ to *λ*_*i*+1_ − 1. Note that we only store the starting label for each group, which requires *O*(Λ) memory.

### 2.3 Intra Edge Generation

In the case of generating any intra edge *e* = (*u*, *v*), where *u*, *v* ∈ *V*_*i*_, the edge (*u*, *v*) is created with probability $p_{u,v}=\frac{b_{u}b_{v}}{S}=\frac{d_{i}^{2}}{S}$ , since *b*_*u*_ = *b*_*v*_ = *d*_*i*_. Notice that, for all pairs of *u*, *v* ∈ *V*_*i*_, the probabilities *p*_*u*,*v*_ are equal. Thus generating the intra edges in *V*_*i*_ is equivalent to generating an Erdős-Rényi (ER) random graph *G*_*i*_(*n*_*i*_, *p*_*i*_) with *n*_*i*_ = |*V*_*i*_| and $p_{i}=\frac{d_{i}^{2}}{S}$ . The ER model *G*(*n*, *p*) generates a random graph with *n* vertices where each of $\frac{n\left(n-1\right)}{2}$ possible potential edges is selected and added to the generated graph with probability *p*. We generate the intra edges on *V*_*i*_ for all *i* by generating ER random graphs $G\left(n_{i},\frac{d_{i}^{2}}{S}\right)$ .

A simple algorithm to generate a random graph *G*(*n*, *p*) is as follows: for each of the $\frac{n\left(n-1\right)}{2}$ potential edges, toss a biased coin and select the edge with probability *p*. As an improvement over this scheme, an efficient algorithm for the ER model based on an edge-skipping technique is available (Batagelj and Brandes, 2005), which we borrow to generate the inter edges for each group.

**FIGURE 2:**
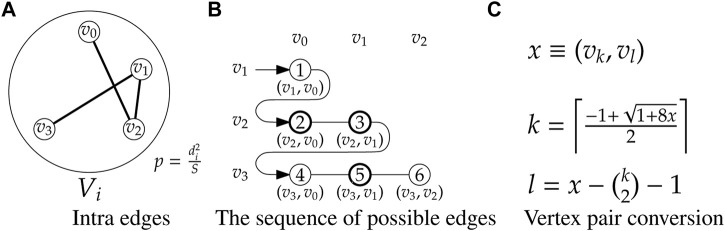
Illustration of intra edge sequences with a group *V*_*i*_ using G(n,p) model with *n*_*i*_ = 4 and $p=\frac{d_{i}^{2}}{S}$ .

For each group *V*_*i*_, to generate the intra edges as an ER random graph $G\left(n_{i},\frac{d_{i}^{2}}{S}\right)$ , we apply the edge skipping technique on the sequence of all potential edges. To save memory space, we avoid creating explicit sequence of the edges. Instead, the edges are represented by a set of consecutive integers 1, 2, …, *M*_*i*_, where Mi=|Vi|2=ni2 , following a lexicographic order of the edges as shown in Figures 2A,B. We select a subset of the integers from 1, 2, …, *M*_*i*_ by applying the skipping technique with the probability $p=\frac{d_{i}^{2}}{S}$ as follows. Let *x* be the last selected edge (initially *x* = 0). The skip length *ℓ* is computed as $\ell =\left\lfloor \frac{\log r}{\log\left(1-p\right)}\right\rfloor$ , where *r* ∈ (0, 1] is a uniform random number. The next selected edge is given by *x* ← *x* + *ℓ* + 1. The selected edge number *x* is converted into an edge using the equations shown in Figure 2C. This process is repeated until *x* ≥ *M*_*i*_.

### 2.4 Inter Edge Generation

**FIGURE 3:**
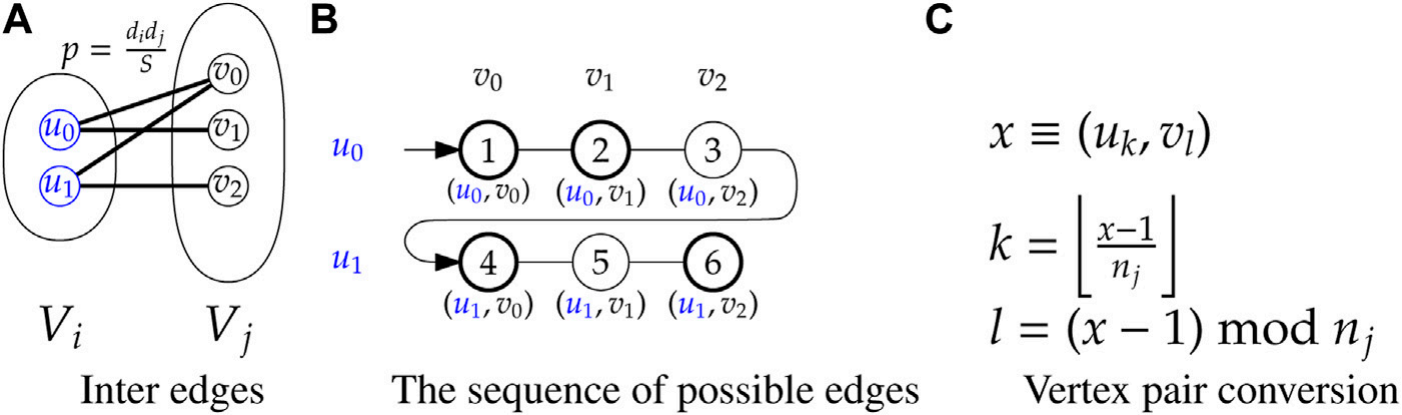
Illustration of inter edge sequences between groups *V*_*i*_ and *V*_*j*_ with *n*_*i*_ = 2 and *n*_*j*_ = 3.

For generating all inter edges, consider any two groups *V*_*i*_ and *V*_*j*_. Given any *u* ∈ *V*_*i*_, *v* ∈ *V*_*j*_, the edge (*u*, *v*) is created with probability $p_{u,v}=\frac{d_{i}d_{j}}{S}$ . Note that for all pairs of *u* ∈ *V*_*i*_, *v* ∈ *V*_*j*_, the probabilities *p*_*u*,*v*_ are equal. Therefore, generating the inter edges between *V*_*i*_ and *V*_*j*_ is equivalent to generating a random bipartite graph (Shang, 2010) formed by two columns of vertices, each with *n*_*i*_ and *n*_*j*_ vertices respectively, and with an edge probability equal to $p=\frac{d_{i}d_{j}}{S}$ (see Figure 3A).

The edge skipping technique is also applied here to generate the inter edges using the random bipartite model (Figure 3A). In this case, the potential edges are represented by consecutive integers 1, 2, …, *M*_*ij*_, where *M*_*ij*_ = |*V*_*i*_‖*V*_*j*_| = *n*_*i*_ *n*_*j*_ (Figure 3B). Next, the edge skipping technique is applied on this sequence with probability $p\;=\;\frac{d_{i}d_{j}}{S}$ . The selected numbers *x* are converted to the edges using the equations shown in Figure 3C.

## 3 Graphics Processing Units-Based Design and Implementation

In this section, we will describe the details of the algorithmic design and implementation for efficient execution on a GPU.

### 3.1 Task Definition and Identification

To generate the whole graph, all possible intra and inter group edges need to be visited, as previously outlined. Note that there are Λ intra edge groups and Λ2=Λ(Λ−1)2 inter edge groups that need to be evaluated. Therefore, there are a total of $\tau =\frac{\Lambda \left(\Lambda +1\right)}{2}$ such groups. Let $T_{i,j}$ represent the task of generating edges between groups *V*_*i*_ and *V*_*j*_, where $d_{i},d_{j}\in D$ . When *i* = *j*, task $T_{i,i}$ generates intra edges; otherwise $T_{i,j}$ produces inter edges. Each edge generation task has a computational cost. Let *c*_*i*,*j*_ be the computational cost of executing task $T_{i,j}$ defined as:

$$c_{i,j}=\alpha +\beta m_{i,j}=\left\{\begin{array}{cc} \alpha +\beta \frac{n_{i}\left(n_{i}-1\right)}{2}\frac{d_{i}^{2}}{S},\; & i=j\\ \alpha +\beta n_{i}n_{j}\frac{d_{i}d_{j}}{S},\; & i\neq j, \end{array}\right.$$ (2)

where, *α* is the fixed cost of time required to initialize a task, *β* is the time to evaluate the generation of an edge, and *m*_*i*,*j*_ is the expected number of edges evaluated by task $T_{i,j}$ .

**FIGURE 4:**
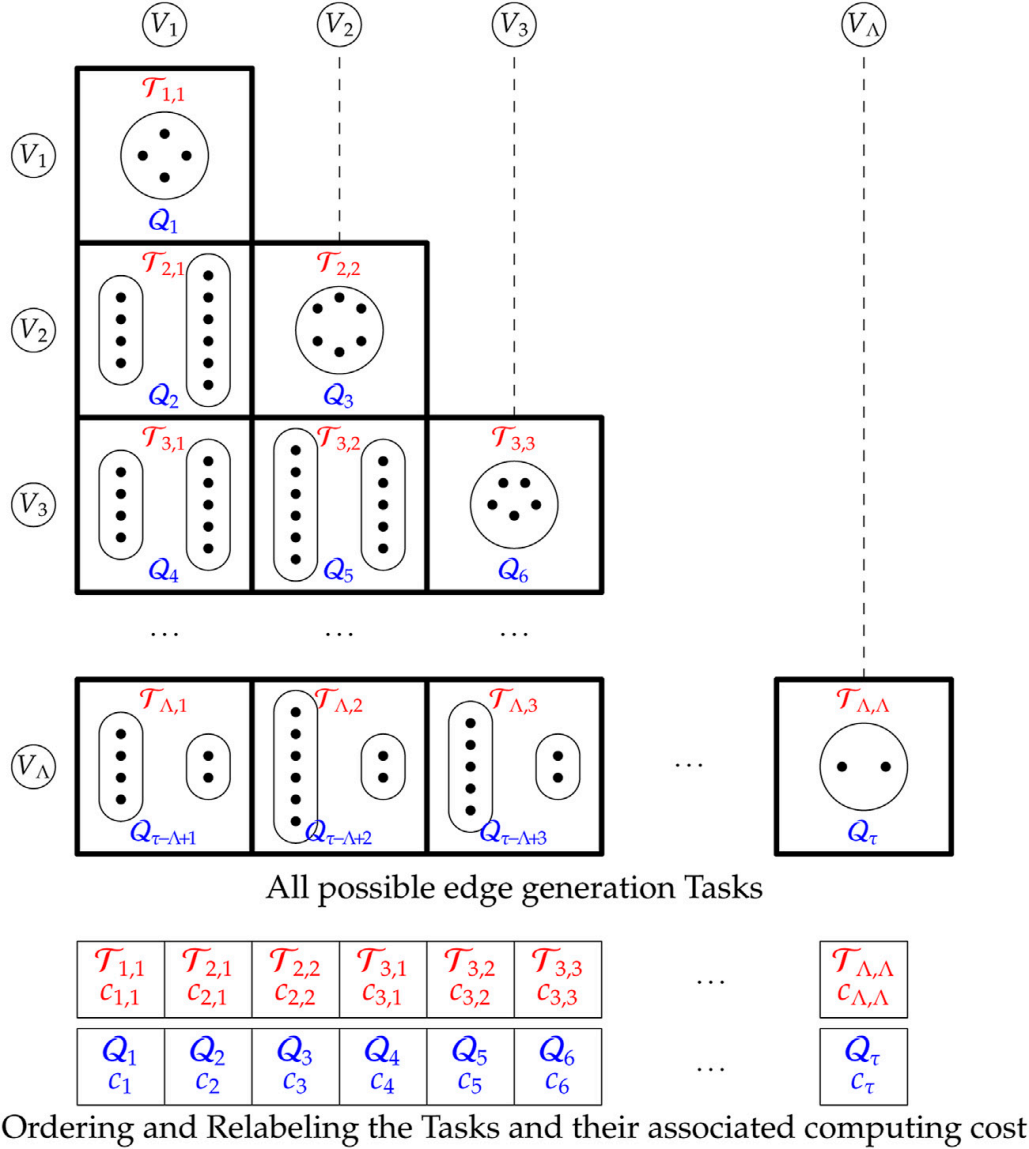
Listing of edge generation tasks $T_{i,j}$ and relabeling to $Q_{x}$ .

To simplify the discussion and implementation, a task $T_{i,j}$ is relabeled from two indices (*i*, *j*) to a single *task number* *x* denoted by $Q_{x}$ , where $x=\frac{i\left(i-1\right)}{2}+j$ . Let *c*_*x*_ be the computing cost of task $Q_{x}$ , that is, *c*_*x*_ = *c*_*i*,*j*_ for the original task $T_{i,j}$ . A visual depiction of the tasks is shown in Figure 4.

The relabeled task $Q_{x}$ can be converted to the original label $T_{i,j}$ using the functions:

$$TASK\text{\_}TO\text{\_}IJ\left(x\right)=\left(i,j\right)\equiv \left(\lceil \frac{-1+\sqrt{1+8x}}{2}\rceil ,x-\frac{i\left(i-1\right)}{2}\right).$$ (3)

### 3.2 Graphics Processing Units Implementation of Intra and Inter Edge Generation Kernels

The GPU implementation is achieved in terms of functions called “kernels” that are launched from the CPU and executed on the GPU. The kernels for the intra– and inter– groups are presented in Algorithm 1. The kernels Kernel-Intra and Kernel-Inter execute the edge generation tasks for intra edges and inter edges, respectively.

**ALGORITHM 1:** GPU Kernels for Generating Edges using Edge Skipping.

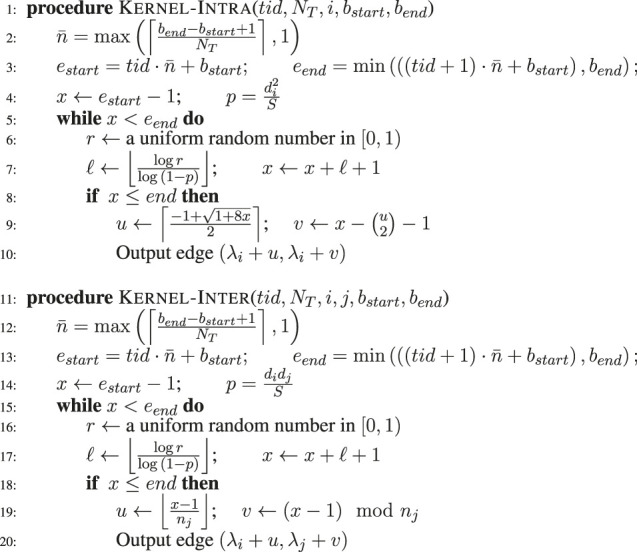

Kernel-Intra uses the following parameters: a thread identifier (*tid*), the number of GPU threads (*N*_*T*_), a group index (*i*), a starting edge index (*b*_*start*_), and an ending edge index (*b*_*end*_) to process the task $T_{i,i}$ . As described in (Alam et al., 2016), any task $T_{i,i}$ can be divided into an arbitrary number of sub-tasks for the ni2 potential edges. Here, 1≤bstart≤bend≤ni2 represent the starting and ending potential edge candidate sequence of a sub-task. Next, the sub-task is processed by *N*_*T*_ concurrent GPU threads with $\bar{n}$ potential edge candidates processed per GPU thread. The variables *e*_*start*_ and *e*_*end*_ denote the starting and ending potential edge candidates of the sub-task for each individual threads, respectively. Using a random number *r* ∈ (0, 1], the skip length *ℓ* is computed in line 7. For computing the skip length, the probability is computed in line 4 as per the CL model formulation. The next selected edge *x* is computed in line 7 and converted to edge (*u*, *v*) in line 9. In line 10, the edge thus generated is represented by (*u*, *v*) ≡ (*λ*_*i*_ + *u*, *λ*_*j*_ + *v*) for the whole graph.

Kernel-Inter uses the following parameters: the thread identifier (*tid*), the number of GPU threads (*N*_*T*_), two group indices (*i*, *j*), a starting edge index (*b*_*start*_), and an ending edge index (*b*_*end*_) to process the task $T_{i,j}$ . The task $T_{i,i}$ can be divided into an arbitrary number of sub-tasks between 1 and *n*_*i*_ *n*_*j*_. Here, 1 ≤ *b*_*start*_ ≤ *b*_*end*_ ≤ *n*_*i*_ *n*_*j*_ represent the range from starting to ending edge indices of work for a sub-task. Similar to the intra kernel, the sub-task is processed by *N*_*T*_ concurrent GPU threads with $\bar{n}$ potential edges processed per GPU thread. The kernel is quite similar to the kernel Kernel-Intra except for the probability (*p*) calculation and edge conversion.

### 3.3 Scheduling the Graphics Processing Units-Based Execution of Edge Generation Tasks

With this task organization, the challenge in efficient network generation becomes that of efficiently scheduling the bag of edge generation tasks $Q=\left\{Q_{i}\right\}$ onto the GPU processing elements. Note that each $Q_{i}$ is either an intra-edge generator or inter edge generator, and the number of edges generated within each task is not uniform.

A GPU consists of many streaming multiprocessors (SMs), each of them consisting of multiple streaming processors (SP) or cores on which GPU threads execute the application’s kernels. GPU threads are organized in a two-level hierarchy of a grid of blocks. A block consists of a specified number of GPU threads, typically up to 1,024 threads. A grid consists of many blocks typically up to 2^31^ − 1 blocks. Each block is executed by one SM and cannot be dynamically migrated to other SMs in the GPU. When a block is executed on an SM, all the GPU threads within the block are executed concurrently. A single SM can run several concurrent blocks depending on the hardware resources available.

To process the set of edge generation tasks Q we designed and implemented the following three schemes to launch, schedule, and execute the tasks on the GPU:

1. GPU Dynamic: Dynamic Asynchronous Kernel Launch
2. GPU Static: Static Kernel Launch, and
3. GPU Static LB: Static Kernel Launch with Load Balanced Cost Partitioning.

#### 3.3.1 Graphics Processing Units Dynamic: Dynamic Asynchronous Kernel Launch

In the dynamic launch scheme, every individual task is launched independently and asynchronously on the GPU and is allowed to utilize the entire GPU resources to generate the edges assigned to the task. As the edge generation tasks are arbitrarily divisible as previously described (Alam et al., 2016), each edge generation task is divided into multiple blocks and threads. Algorithm 2 shows the dynamic launching scheme. The CPU code launches the GPU kernels (line 6 and 9) based on type of the task (whether it is an intra-edge or inter edge generator). To distribute the task across multiple concurrent threads and blocks, we use a fixed GPU threads, *N*_*T*_ per block. The number of GPU blocks is then determined by *N*_*B*_ (line 5 and 8) based on the computational cost, which in this case is assumed to be the expected number of edges to be generated.

The specific choice of *N*_*T*_ and *N*_*B*_ varies with the specific GPU hardware being used. For instance, with the GPU used in our experiments, the runtime performance profile from the NVIDIA profiling tool nvprof shows the number of registers per kernel to be 62. Among the choices for *N*_*T*_, 512 threads was observed to provide the best performance, while 1,024 threads is the upper limit considering the number of registers per kernel. The hardware limits set by the graphics card provide up to 128 registers per thread, and the maximum number of registers per block is 65536. This would imply that our kernel can be executed with *N*_*T*_ ≤ 1024 threads per block without exceeding the hardware register limits. In practice, we found that the *N*_*T*_ = 512 thread-execution achieves a slightly higher runtime performance than the 1024-thread execution. For this reason, we set the number of threads to 512. Similarly, we used a block size *N*_*B*_ of 20000. Although this setting can be varied by problem size, we found experimentally that this value provides the best runtime performance across different networks. However, clearly this choice of block size will in general vary with the specific GPU card, as it does for many GPU applications.

Note that, as multiple GPU kernels are launched asynchronously by the CPU host, we need to synchronize the GPU after a number of kernels (32 in our experiments) are launched, to avoid any scheduling overhead. Between synchronization points, multiple independent GPU streams are used to avoid needless ordering among the kernels, so that the GPU can execute all scheduled tasks whenever hardware resources become available.

**ALGORITHM 2:** Dynamic Kernel Launch.

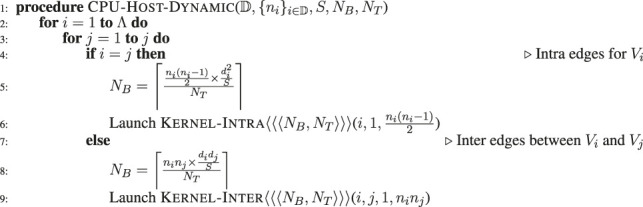

Although dynamic launching of kernels is simple to implement and effective in many applications, there are potential disadvantages in the context of graph generation. One of the main problems is that, due to the non-uniformity of work across tasks, the number of edges generated per thread can be low, which makes the thread execution overhead high. Another issue is that the distribution of computation cost across the tasks can be skewed; moreover, the number of tasks can become very high (as in the case of large values of Λ). Therefore, the dynamic scheme can incur significant kernel launch and scheduling overheads. For some of the tasks with a low number of expected edges, the overhead of kernel launch is too high to offset the computational gain from launching that task on the GPU. Note that the problem is magnified when the $\frac{\Lambda^{2}}{m}$ is higher, as observed later in the experimental evaluation.

#### 3.3.2 Graphics Processing Units Static: Static Kernel Launch

In this static kernel launching scheme, we use a predefined number of blocks (*N*_*B*_) and threads per block (*N*_*T*_) rather than computing those dynamically based on the task workload. This approach is shown in Algorithm 3. There is only one kernel launch call as shown in line 11 using a predefined set of values for *N*_*B*_ and *N*_*T*_. Based on the insight that better performance can be achieved by assigning more work to each thread (Volkov, 2010), this scheme aims to allocate more work to every thread by distributing the edge generation tasks evenly among the threads and setting appropriate values of *N*_*B*_ and *N*_*T*_. The *τ* edge generation tasks are distributed evenly among the *N*_*B*_ GPU blocks. Therefore, each GPU block executes $\bar{\tau }=\frac{\tau }{N_{B}}$ edge generation tasks (line 2). A GPU block denoted by *bid* executes tasks from *t*_*start*_ to *t*_*end*_ (line 3). Each edge generation task is executed by the *N*_*T*_ concurrent threads on the GPU based on the type of the task (intra or inter) on lines 7 and 9.

**ALGORITHM 3:** Static Kernel Launch.

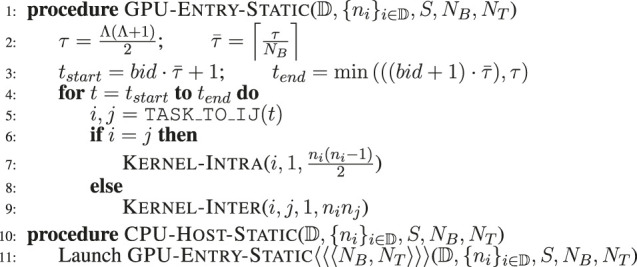

A potential issue with the static kernel launch is that it does not specifically account for the computational cost for the processing and scheduling of the edge generation kernels. As the distribution of the computational cost of the tasks can be potentially skewed, the expected number of edges produced by each GPU block may vary significantly. Therefore, some GPU blocks may take significantly longer amounts of time compared to other GPU blocks. Note that the GPU does not guarantee the concurrent execution of all GPU blocks at the same moment on the GPU device; rather, it executes a limited number of GPU blocks concurrently based on the available number of SMs. Once one GPU block finishes execution, it is replaced by another GPU block on the SM. Therefore, to get around this issue, we use a sufficiently large number of GPU blocks *N*_*B*_ such that longer GPU blocks can continue to execute on the GPU while shorter GPU blocks finish execution on the GPU and be replaced by other unprocessed blocks.

#### 3.3.3 Graphics Processing Units Static LB: Static Kernel Launch With Load Balancing

To address the problem of skewed distribution of computational costs among the edge generation tasks, we designed another algorithmic variant that starts with the static launch scheme and adds the Uniform Cost Partitioning (UCP) approach presented in our previous CPU-based generator (Alam et al., 2016). In this case, the task boundaries (*t*_*start*_ and *t*_*end*_) are determined using a uniform distribution of the computational cost. Each block executes the tasks based on the task boundaries and, therefore, each block has nearly the same expected computational cost as other blocks.

## 4 Performance Study

In this section, we present an evaluation of our generator and its performance by an experimental study and analysis, in terms of speed of generation and the quality of the output degree distribution. The evaluation is performed using a range of real-world input degree distributions. For the purposes of time analysis, assuming a streaming mode of usage of the graphs, the memory I/O time to write the graph is not included.

### 4.1 Hardware and Software

All experiments are executed on a computer consisting of Intel(R) Xeon(R) Silver 4110 CPU with a 2.1 GHz clock speed and 256 GB system memory. The machine also incorporates an NVIDIA Tesla V100 GPU with 16 GB memory. The operating system is Ubuntu 20.04 LTS. All software on this machine was compiled with GNU gcc 7.4.0 with optimization flags -O3. The CUDA compilation tools V11 were used for the GPU code along with the nvcc compiler.

### 4.2 Input Degree Distribution

For the purposes of testing our algorithms, we used degree distributions from publicly available real-world networks (Boldi and Vigna, 2004; Boldi et al., 2008; Kwak et al., 2010). The networks and their original and coarsened measures are listed in Table 1 (degree distribution coarsening will be discussed in the following subsections). The networks vary in the number of vertices, edges, and the number of unique degrees. The number of vertices vary from 1.98 million (Hollywood) to 1.07 billion (EU-2015), and the number of edges vary from 49 million (LiveJournal) to 69 billion (FB-Current). Similarly, the sizes of the degree distributions also vary widely. Importantly, the ratio $\frac{\Lambda^{2}}{m}$ has significant bearing on the GPU-based generation because it determines the amount of variation of workload among the tasks. The lower the ratio, the more uniform the workload distribution, the greater the load balance, and the lower the divergence among GPU threads, as will be seen later in the runtime performance variation for the networks.

**TABLE 1:** Original and coarsened input degree distributions of the test networks.

Network			Original		Coarsened	
Name	Vertices *n*	Edges *m*	Λ	$\frac{\Lambda^{2}}{m}$	Λ*	$\frac{\Lambda^{*2}}{m}$
LiveJournal	4,889,483	49,520,700	1,877	0.0711	1,788	0.0646
Hollywood	1,977,070	113,906,622	5,361	0.2523	3,775	0.1251
Twitter	40,603,079	1,153,360,000	14,844	0.1910	12,876	0.1437
Friendster	65,608,366	1,806,065,000	3,148	0.0055	2,608	0.0038
WebGraph	445,394,551	3,140,069,461	26,890	0.2303	16,018	0.0817
UK-Union	127,980,140	4,462,300,000	39,826	0.3554	23,191	0.1205
EU-2015	1,070,557,254	45,896,130,800	71,218	0.1105	27,723	0.0167
FB-Current	721,094,633	69,014,500,000	4,999	0.0004	4,999	0.0004

### 4.3 Generating Networks With Original Input Degree Distribution

Table 2 shows the time taken for the generation of the networks from the test network degree distributions shown in Table 1. The time taken using a single CPU core is compared with the time taken using each of our three algorithmic variants on the GPU. It is observed that the GPU execution is faster than CPU execution across the board. The time varies due to the size of the network and the efficiency of the algorithm.

**TABLE 2:** Network generation time for original input degree distributions.

	1 CPU core	GPU runtime (s)			CPU-GPU
Network	Runtime (s)	Static	Static LB	Dynamic	Transfer (s)
LiveJournal	2.00	0.03	0.03	3.50	0.150
Hollywood	5.26	0.20	0.21	29.42	0.154
Twitter	48.76	1.35	1.44	232.68	0.151
Friendster	66.61	0.14	0.15	11.41	0.155
WebGraph	136.04	4.36	4.63	841.12	0.151
UK-Union	217.01	8.88	9.46	1,880.50	0.151
EU-2015	1,855.98	34.07	35.88	4,030.49	0.156
FB-Current	2,518.03	1.37	1.36	30.60	0.150

**FIGURE 5:**
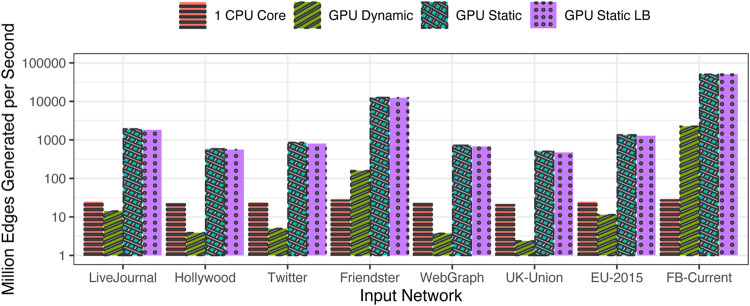
Speed of generation (million edges generated per second) for the original input distributions.

For a more uniform evaluation, the performance is normalized using the metric of millions of events generated on average per second, shown in Figure 5. The largest generation rate is seen in the case of FB-Current network, followed by the Friendster network. Both these networks have a low value of $\frac{\Lambda^{2}}{m}$ , which translates to a large amount of concurrency across tasks. In other words, the number of edges to be generated in each task is so large that the overheads associated with thread launches and task assignment are greatly amortized across the tasks.

### 4.4 Degree Distribution Coarsening

Since the value of $\frac{\Lambda^{2}}{m}$ has a strong bearing on the runtime performance, the original input degree distribution needs to be filtered into another equivalent degree distribution that preserves the quality and shape of the distribution that has fewer bins and hence increases the value of $\frac{\Lambda^{2}}{m}$ .

For this purpose, we designed a degree distribution coarsening method shown in Algorithm 4. This method is based on an intuitive approach as follows.

In large-scale networks, as the number of vertices gets larger, the number of unique degrees also becomes larger. This increases the number groups, Λ. However, we notice from the test networks that there are many unique degrees that are relatively close together, that is, *d*_*i*_ and *d*_*i*+1_ differ only by very small amounts (for example, *d*_*i*+1_ = *d*_*i*_ + 1). When *d*_*i*_ is relatively large (such as *d*_*i*_ = 1,000, it is clear that they can be practically considered equal. However, without any adjustments, the GPU algorithm will consider them as two distinct groups and spend increased amount of computational time in generating many inter edges and correspondingly fewer intra edges. Because intra edge generation is much faster than inter edge generation, and since the number of tasks grows quadratically with the number of groups, the needless distinction between *d*_*i*_ and *d*_*i*+1_ when they are quantitatively close creates a significant runtime overhead. Therefore, in our coarsening method, we coalesce groups whose degrees are numerically close to each other.

Quantitatively, we define a tolerance *δ* such that *d*_*i*_ and *d*_*i*+1_ are coalesced when *d*_*i*+1_ ≤ (1 + *δ*)*d*_*i*_, where a specific value of *δ* is chosen for a given network, 0 < *δ* ≤ 1 (for EU-2015, *δ* = 0.02, for example). The coalesced groups (*d*_*i*_, *n*_*i*_) and (*d*_*i*+1_, *n*_*i*+1_) are replaced by a composite group $\left(\bar{d_{i}},\bar{n_{i}}\right)$ , where $\bar{d_{i}}=xyz$ and $\bar{n_{i}}=xyz$ . Note that, after coalescing, the groups are shifted left and reduced in number by one, that is, (*d*_*i*_, *n*_*i*_) is replaced by $\left(\bar{d_{i}},\bar{n_{i}}\right)$ , and (*d*_*i*+1_, *n*_*i*+1_) is removed from the distribution.

At the lower end of the degree distribution, the distinction between the degrees would be important to preserve, even if they are relatively close together. Therefore, we define a lower threshold *d*_*cut*_ below which we do not alter the degree distribution. In other words, all *d*_*i*_ ≤ *d*_*cut*_ of the original input distribution are preserved unmodified in the coarsened input distribution.

**ALGORITHM 4:** Input Degree Distribution Coarsening Algorithm.

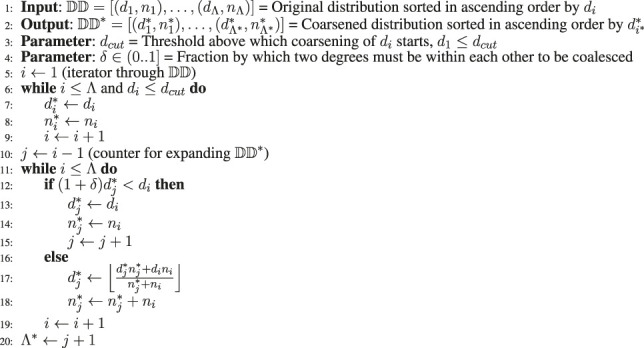

**FIGURE 6:**
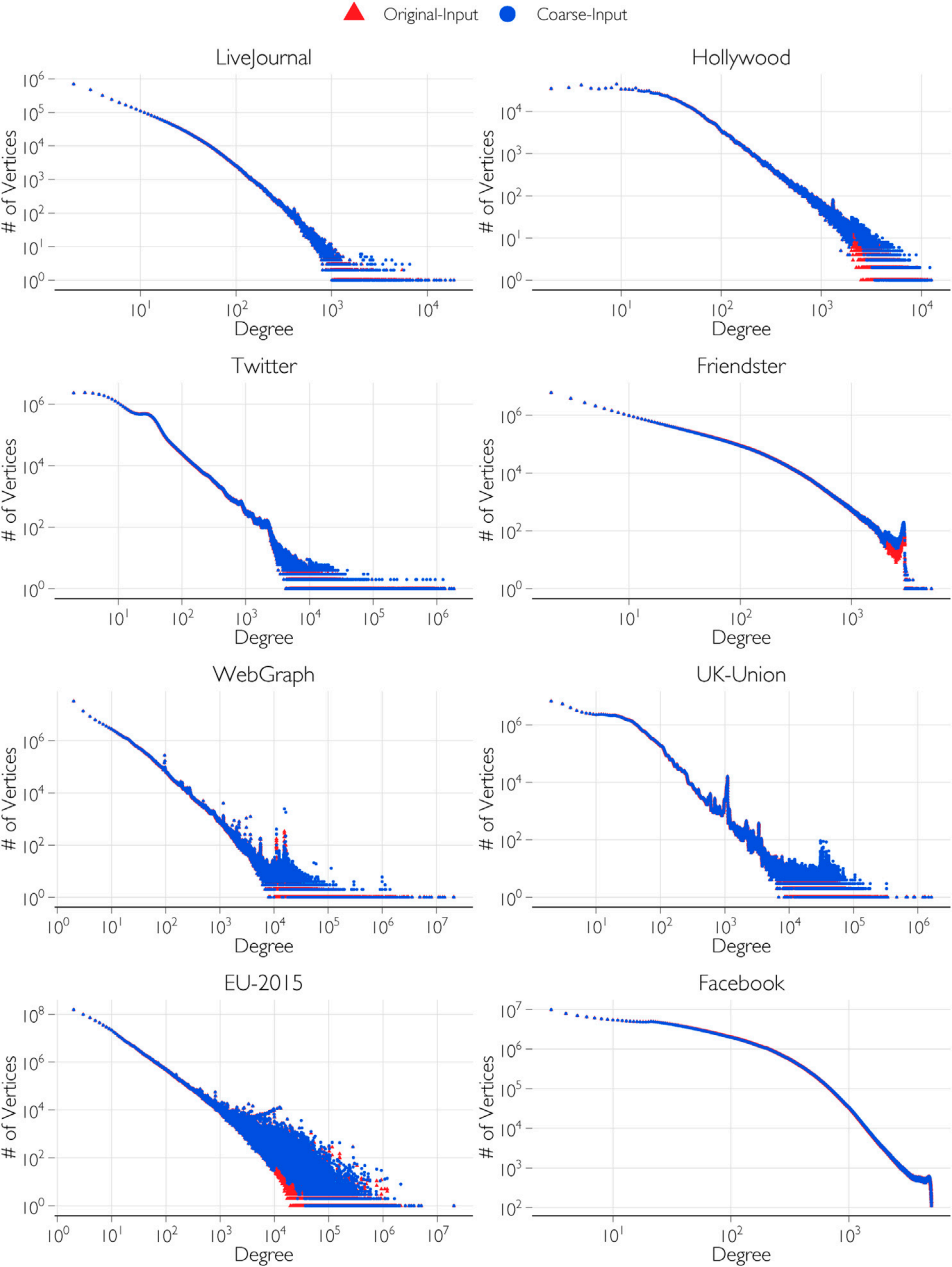
Comparison of the original input degree distributions and coarsened input degree distributions.

**FIGURE 7:**
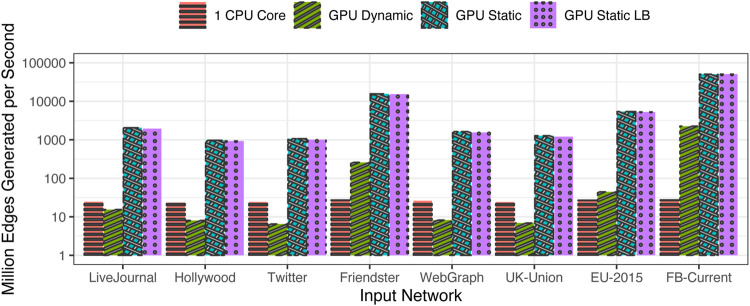
Million edges generated per second for the coarsened distribution.

In Figure 6, the original input degree distributions are compared with the coarsened input distribution by plotting the number of vertices for each degree contained in the distribution. The plots show a close match of the distribution even while reducing the number of groups and increasing the group sizes. In Table 1, the number of groups, Λ, of the original input distribution is compared with the reduced number of groups, Λ*, after coarsening the distribution. For networks such as UK-Union and EU-2015, the improvement in the smoothness is significant. This is evident both in the reduction in the number of groups from Λ to Λ*, and corresponding reduction from $\frac{\Lambda^{2}}{m}$ to $\frac{\Lambda^{*2}}{m}$ . Because the number of tasks increases as the square of the number of groups, the reductions from coarsening results in significantly fewer and more uniformly loaded tasks for execution on the GPU threads. This in turn results in reduction in the overheads for thread launch and also reduces the unevenness in the amount of work per task as shown in Figure 7.

### 4.5 Generating Networks With Coarsened Input Degree Distribution

Table 3 shows the time taken for the generation of the networks from the test input degree distributions of Table 1 after applying the coarsening algorithm on them. Compared to the time taken with the original input distributions, the time taken with the coarsened input distributions is significantly reduced for some networks. On the CPU, the time is largely unchanged, but the time on the GPU is significantly reduced. This is particularly pronounced for EU-2015 in which the coarsening significantly reduced the number of groups from 71,218 to 27,723 even while maintaining roughly the same distribution as seen in Figure 6. Similarly, for UK-Union, the number of groups is reduced by coarsening from 39,826 groups to 23,191.

**TABLE 3:** Network generation time from coarsened input degree distribution.

	1 CPU core	GPU runtime (s)			CPU-GPU
Network	Runtime (s)	Static	Static LB	Dynamic	Transfer (s)
LiveJournal	2.01	0.02	0.03	3.21	0.154
Hollywood	4.85	0.12	0.12	14.20	0.153
Twitter	47.32	1.08	1.14	177.29	0.156
Friendster	66.28	0.12	0.12	6.97	0.151
WebGraph	120.11	1.93	2.02	380.90	0.152
UK-Union	184.26	3.50	3.71	645.57	0.156
EU-2015	1,707.34	8.49	8.69	1,030.88	0.154
FB-Current	2,518.03	1.37	1.36	30.60	0.156

**FIGURE 8:**
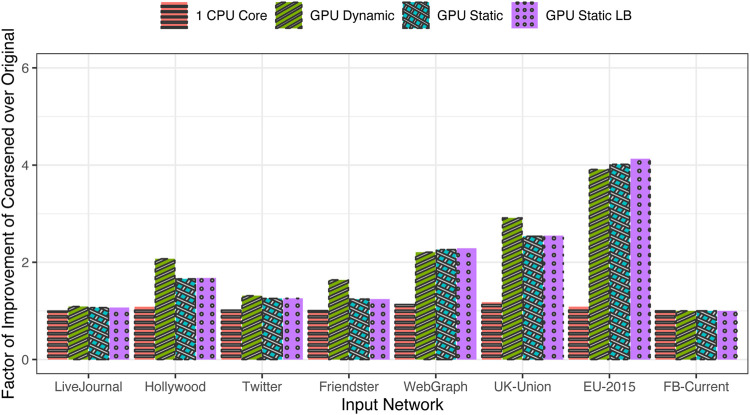
Factor of improvement in run time using coarsened versus original distribution.

The factor of improvement in generation time when moving from the original input distributions to the coarsened input distributions is shown in Figure 8. It is seen that the run time is largely unaffected on the CPU because the single-core execution is largely insensitive to the workload variation among the tasks; the large amount of CPU caching capacity works well to smooth out most such variations in the working set of the application. However, the gains are most prominent on the GPU, especially for those distributions that exhibit wide variance among the task workloads. As expected, the largest gains are observed for the EU-2015 network, and the next best is observed for the UK-Union and WebGraph data sets. The speed is nearly doubled in the case of Hollywood and nearly quadrupled in the case of EU-2015. Also, speed is more than doubled for WebGraph and UK-Union. However, in the others, coarsening does not lead to any appreciable reductions in Λ and consequently does not appreciably improve the generation time.

**FIGURE 9:**
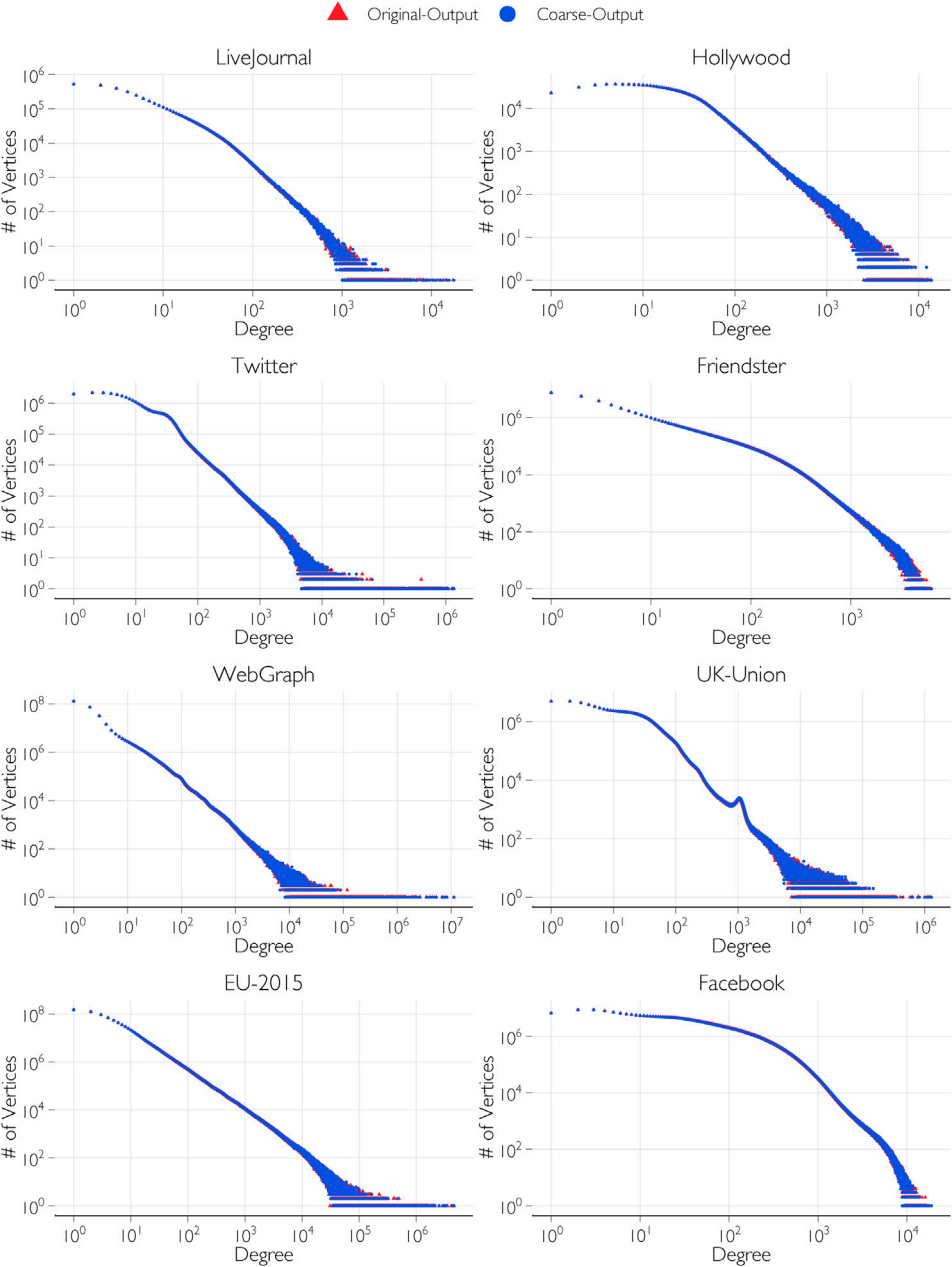
Validating the output degree distribution from coarsened input distributions by comparison with that from original input distributions.

In Figure 9, the output degree distributions of the generated networks are compared between the original and coarsened inputs to validate the output degree distribution. The output distributions show very close match between the output networks generated from the original input distributions and the output networks generated from the coarsened input distributions. The closeness of the distributions of the generated networks to the input distributions have been quantitatively verified using the Kullback-Leibler (K-L) divergence metric (also called relative entropy). For example, in the case of the Twitter network, the difference is 0.11% and in the case of the UK-Union network, the difference is 0.24%. These are taken as acceptable differences due to the randomness of the generated networks. These are documented in our previous work with CPU-based algorithms (Alam and Khan, 2015; Alam et al., 2016).

### 4.6 Graphics Processing Units Performance Metrics

In this section, we measure the performance of the GPU kernels for network generation. The performance metrics was collected using the native NVIDIA Profiler (nvprof) using various performance metrics. The summary of the results is presented in Table 4. For this profile, we use the static version of the algorithm, executed with 512 threads per block. The number of blocks is set to 20,000, which delivers the highest performance. The profiles are collected for the smallest and largest networks, namely, LiveJournal and FB-Current, respectively.

**TABLE 4:** Performance metrics of the kernels using the NVIDIA Profiler nvprof.

Metrics	Smallest graph	Largest graph
	(LiveJournal)	(FB-current)
Registers/thread	62	62
Theoretical occupancy	50.00%	50.00%
Achieved occupancy	39.19%	45.70%
SM efficiency	99.66%	99.65%
Branch efficiency	96.48%	89.60%
Warp execution efficiency	89.55%	77.18%

The registers/thread, which is the number of registers used by each kernel executing within one thread, is determined to be 62, which allows our kernel to have 100% theoretical occupancy. Theoretical occupancy is the number of threads in a warp that were executed compared to the maximum number of threads that could be executed. Although our kernels can be launched with 100% occupancy, we use a lower level of 50% theoretical occupancy, which gives better runtime performance due to other caching effects–this is in line with the insight in the literature on other applications that a lower level of occupancy can increase runtime performance to an extent (Volkov, 2010). Our achieved occupancy (45.70% for the large network) comes close to the theoretical occupancy. The streaming multiprocessor (SM) efficiency, which is the percentage of time the SM is busy doing application’s work, as opposed to scheduling and blocking operations, is very high at 99.6%. Branch efficiency is also observed to be very high (96.48%) on a small network, and fairly high (89.60%) on the largest network.

## 5 Summary and Future Work

We presented a novel GPU-based algorithm for generating large random networks that conform to desired degree distributions provided as input. To our knowledge, this is the first algorithm designed, implemented, and evaluated on GPUs for degree distribution-defined network generation. Three algorithmic variants are presented for execution on the GPU based on the different scheduling strategies for mapping the generation tasks to GPU threads. The algorithms have been implemented on a modern NVIDIA GPU and a detailed performance study has been performed using the degree distributions of a range of test networks containing millions to billions of edges. The effect of task size in terms of the number of edges to be generated is observed to have significant bearing on the performance for some test networks. To further improve the performance of the generator on the SIMT architectures of GPUs, a distribution coarsening method has been designed and implemented, which retains the sizes and quality of the input distributions while generating similar output distributions at an increased rate. The overall network generation rates observed from our performance study exceeds 50 billion edges generation per second, which is among the fastest generation rates reported in the literature using a single desktop computer.

Modern workstations commonly offer more than one GPU connected to the same system. Our algorithm can be extended to exploit the multi-GPU systems by scheduling the tasks across multiple GPUs, which we intend to explore in future work. Similarly, many modern high performance parallel computing systems offer multiple interconnected machines, each containing one or more GPUs. The approach presented here could be extended to such distributed cluster of GPUs for increased scale and speed of network generation.

## Data Availability

Publicly available datasets were analyzed in this study. This data can be found here: Webgraph, https://webgraph.di.unimi.it.
